# Supplementary material for: Transcriptomic Insights into Mechanisms of Early Seed Maturation in the Garden Pea (Pisum sativum L.)
Source: Cells. 2020 Mar 23;9(3):779. doi: 10.3390/cells9030779 (PMC7140803; doi:10.3390/cells9030779)
Supplement: Supplementary file 1 [file cells-09-00779-s001.zip › Supplementary materials/FileS2.zip!/FileS1/Summary Sheets - Modules/Correlation Cluster/Report.pdf]

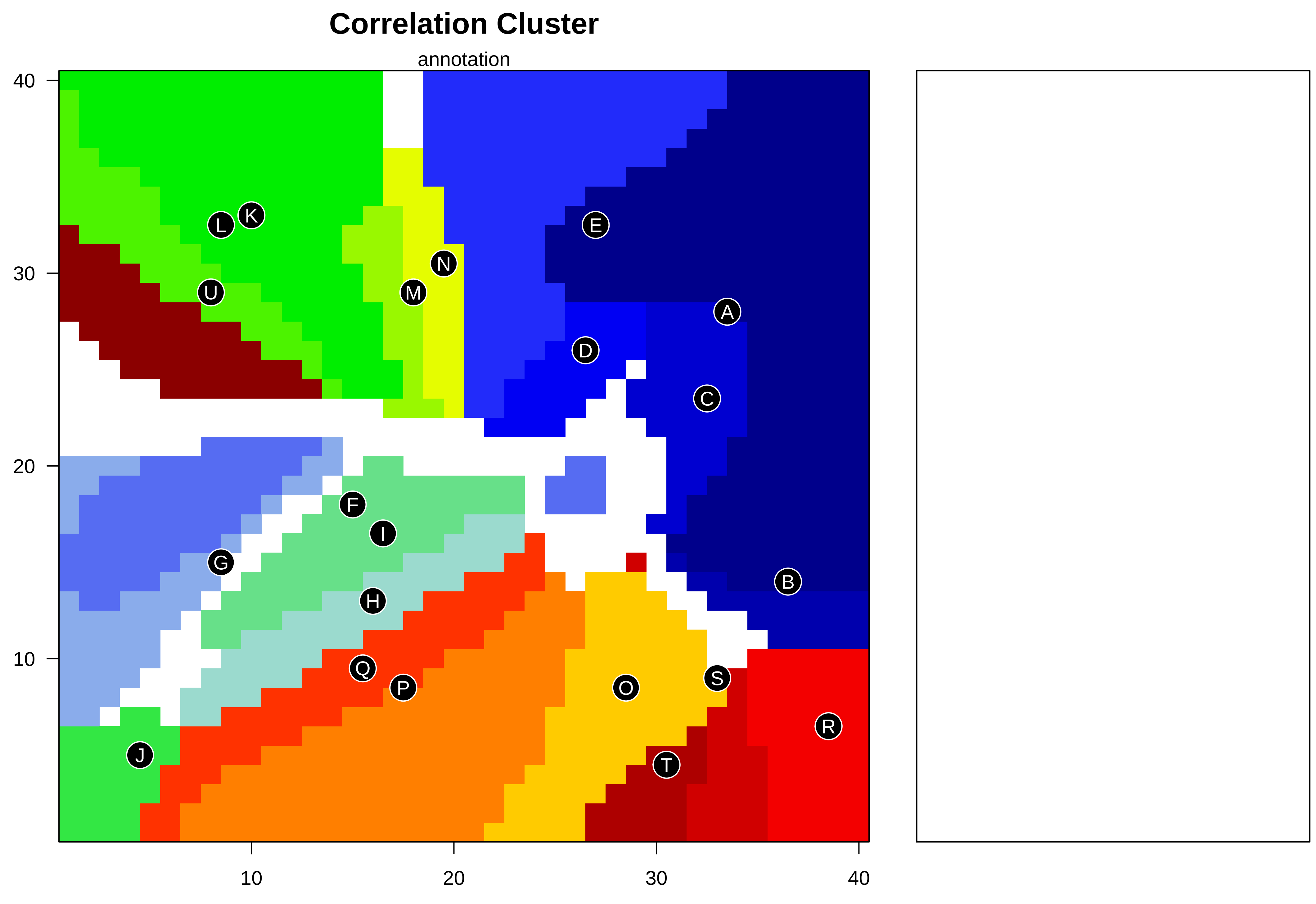
Correlation Cluster
annotation
40
30
20
10
10
20
30
40
A
B
C
D
E
F
G
H
I
J
K
L
M
N
O
P
Q
R
S
T
U

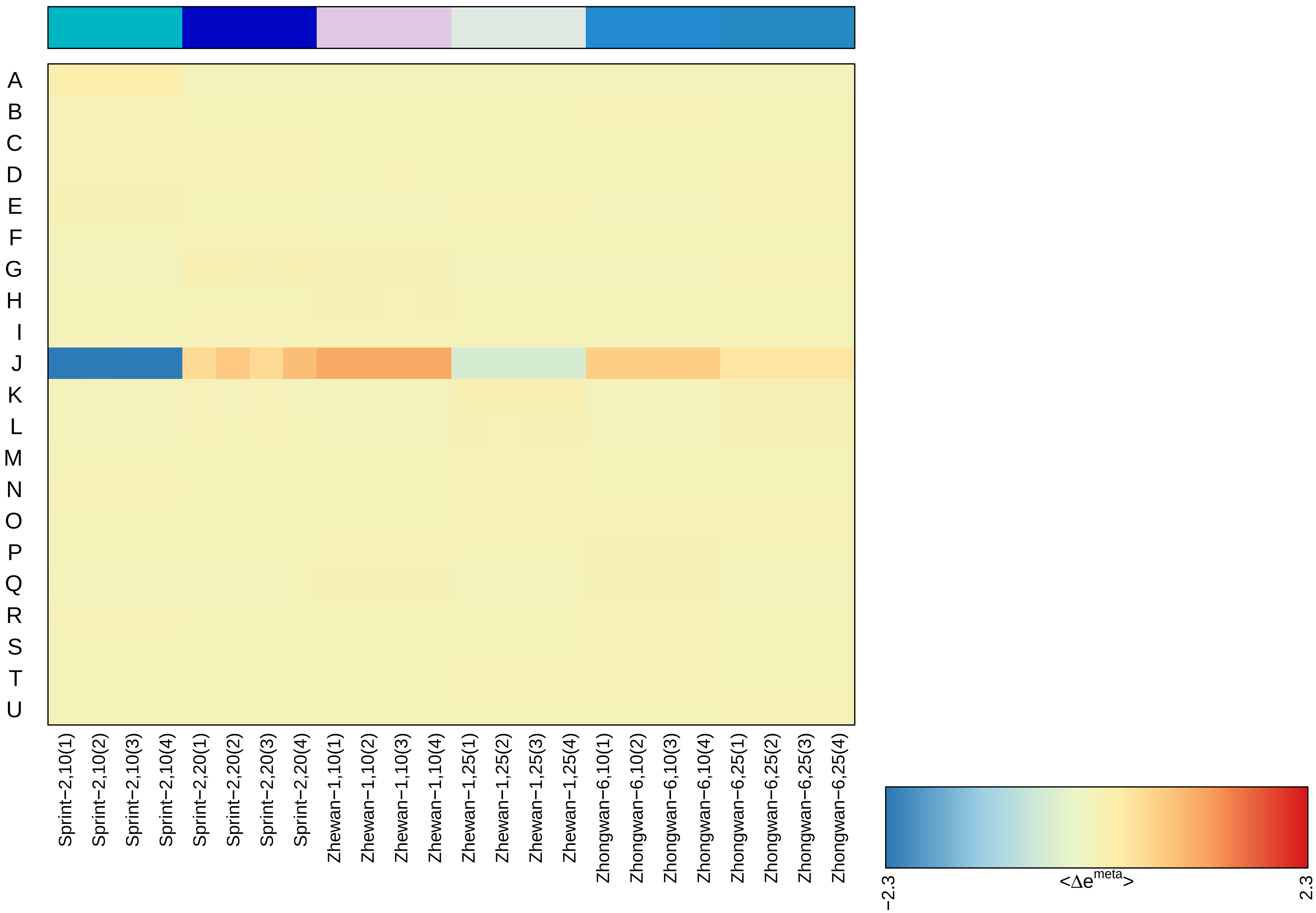
A
B
C
D
E
F
G
H
I
J
K
L
M
N
O
P
Q
R
S
T
U
Sprint−2,10(1)
Sprint−2,10(2)
Sprint−2,10(3)
Sprint−2,10(4)
Sprint−2,20(1)
Sprint−2,20(2)
Sprint−2,20(3)
Sprint−2,20(4)
Zhewan−1,10(1)
Zhewan−1,10(2)
Zhewan−1,10(3)
Zhewan−1,10(4)
Zhewan−1,25(1)
Zhewan−1,25(2)
Zhewan−1,25(3)
Zhewan−1,25(4)
Zhongwan−6,10(1)
Zhongwan−6,10(2)
Zhongwan−6,10(3)
Zhongwan−6,10(4)
Zhongwan−6,25(1)
Zhongwan−6,25(2)
Zhongwan−6,25(3)
Zhongwan−6,25(4)
−2.3
<Δe^meta>
2.3

Correlation Cluster

Spot Summary: A

metagenes = 254
genes = 3240

<r> metagenes = 0.94

beta: r2= 0.06 / log p= −0.62

samples with spot = 0 (0 %)

Overview Map

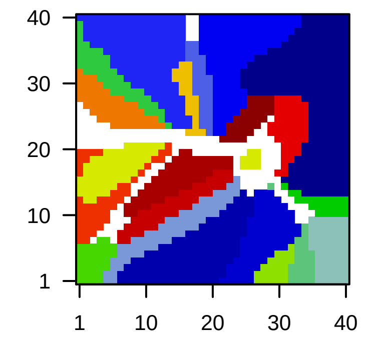

Spot

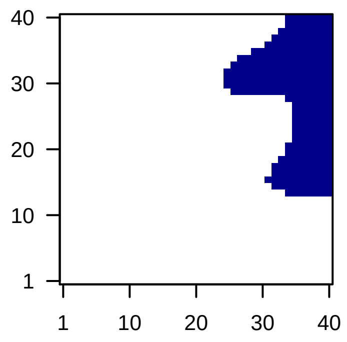

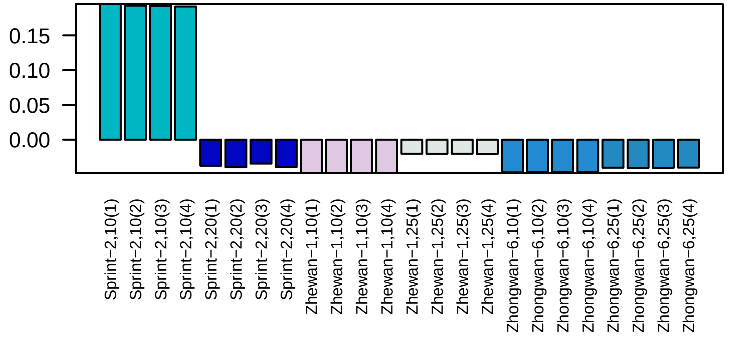

Spot Genelist

Rank	ID	max e	min e	r	Symbol	Description
1	TRINITY_DN7	117.83	−10.22	0.78		
2	TRINITY_DN2	109.03	−18.72	0.93		
3	TRINITY_DN1	36.31	−5.78	0.96		
4	TRINITY_DN8	27.57	−5.74	0.99		
5	TRINITY_DN4	22.28	−3.73	0.99		
6	TRINITY_DN3	14.63	−3.64	0.99		
7	TRINITY_DN3	13.41	−2.83	0.98		
8	TRINITY_DN8	11.22	−3.35	0.98		
9	TRINITY_DN3	11.09	−2.42	0.98		
10	TRINITY_DN2	10.84	−1.3	0.82		
11	TRINITY_DN5	10.56	−2.04	0.95		
12	TRINITY_DN1	10.49	−2.06	0.99		
13	TRINITY_DN3	9.42	−1.6	0.98		
14	TRINITY_DN5	9.26	−1.71	0.69		
15	TRINITY_DN1	8.44	−1.61	0.96		
16	TRINITY_DN1	7.68	−1.68	0.97		
17	TRINITY_DN4	7.37	−1.78	0.97		
18	TRINITY_DN1	7.34	−2.04	0.69		
19	TRINITY_DN7	6.84	−1.21	0.99		
20	TRINITY_DN1	6.54	−1.49	0.99		

Correlation Cluster

Spot Summary: B

metagenes = 22
genes = 449

<r> metagenes = 0.97
<r> genes = 0.68
beta: r2= 0 / log p= −0.01

samples with spot = 0 (0 %)

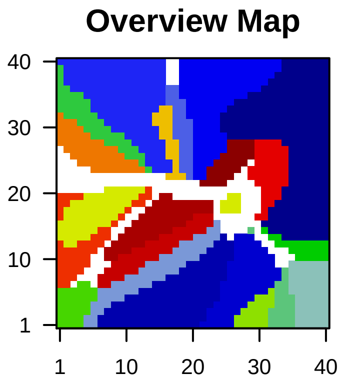

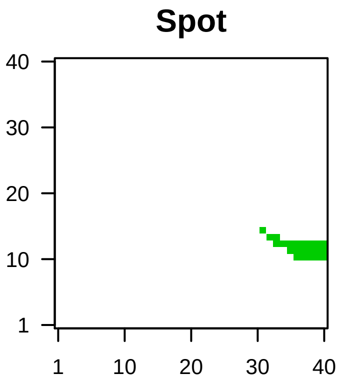

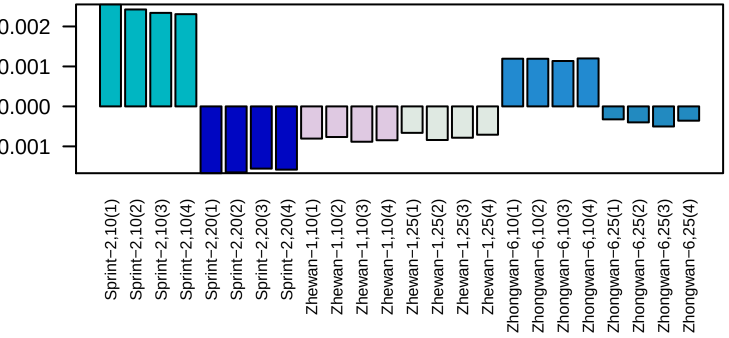

Spot Genelist

Rank	ID	max e	min e	r	Symbol	Description
1	TRINITY_DN3	0.02	−0.02	0.39		
2	TRINITY_DN8	0.02	−0.01	0.81		
3	TRINITY_DN1	0.01	0	0.86		
4	TRINITY_DN5	0.01	−0.01	0.76		
5	TRINITY_DN6	0.01	−0.01	0.55		
6	TRINITY_DN7	0.01	−0.01	0.35		
7	TRINITY_DN1	0.01	−0.01	0.75		
8	TRINITY_DN7	0.01	−0.01	0.61		
9	TRINITY_DN4	0.01	−0.01	0.36		
10	TRINITY_DN6	0.01	−0.01	0.79		
11	TRINITY_DN5	0.01	−0.02	0.54		
12	TRINITY_DN1	0.01	−0.01	0.84		
13	TRINITY_DN5	0.01	−0.01	0.61		
14	TRINITY_DN5	0.01	−0.02	0.65		
15	TRINITY_DN3	0.01	0	0.86		
16	TRINITY_DN1	0.01	−0.01	0.66		
17	TRINITY_DN4	0.01	−0.01	0.51		
18	TRINITY_DN4	0.01	−0.01	0.88		
19	TRINITY_DN8	0.01	−0.01	0.55		
20	TRINITY_DN5	0.01	−0.01	0.66		

Correlation Cluster

Spot Summary: C

metagenes = 47
genes = 823

<r> metagenes = 0.95
<r> genes = 0.65
beta: r2= 0 / log p= −0.05

samples with spot = 0 (0 %)

Overview Map

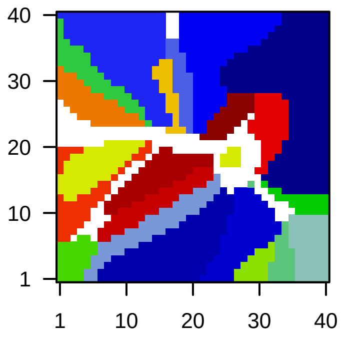

Spot

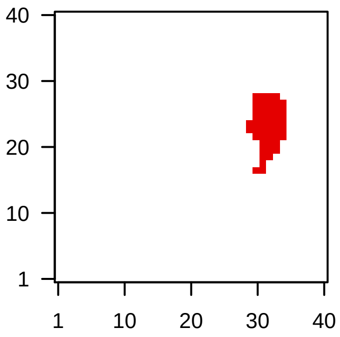

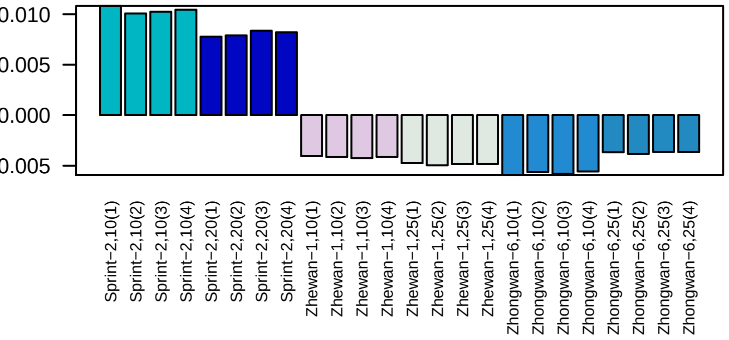

Spot Genelist

Rank	ID	max e	min e	r	Description	Symbol
1	TRINITY_DN9	0.08	−0.05	0.83		
2	TRINITY_DN5	0.08	−0.02	0.82		
3	TRINITY_DN5	0.07	−0.07	0.55		
4	TRINITY_DN3	0.07	−0.04	0.81		
5	TRINITY_DN1	0.07	−0.02	0.57		
6	TRINITY_DN1	0.06	−0.04	0.8		
7	TRINITY_DN2	0.06	−0.04	0.93		
8	TRINITY_DN4	0.05	−0.02	0.95		
9	TRINITY_DN4	0.05	−0.02	0.93		
10	TRINITY_DN6	0.05	−0.05	0.66		
11	TRINITY_DN7	0.05	−0.04	0.85		
12	TRINITY_DN3	0.05	−0.06	0.79		
13	TRINITY_DN9	0.05	−0.02	0.95		
14	TRINITY_DN2	0.05	−0.04	0.91		
15	TRINITY_DN3	0.05	−0.03	0.89		
16	TRINITY_DN1	0.05	−0.03	0.94		
17	TRINITY_DN2	0.05	−0.04	0.86		
18	TRINITY_DN7	0.05	−0.03	0.95		
19	TRINITY_DN4	0.05	−0.02	0.9		
20	TRINITY_DN2	0.05	−0.05	0.49		

Correlation Cluster

Spot Summary: D

metagenes = 31
genes = 461

<r> metagenes = 0.96
<r> genes = 0.57
beta: r2= 0 / log p= −0.04

samples with spot = 0 (0 %)

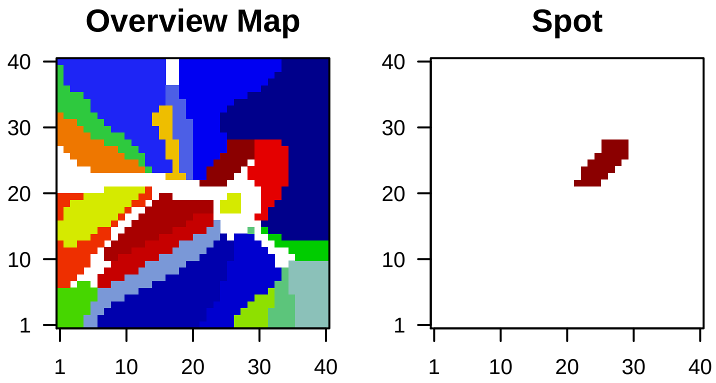

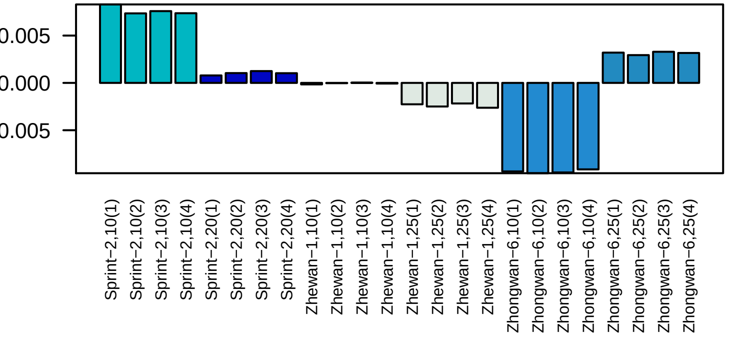

Spot Genelist

Rank	ID	max e	min e	r	Symbol	Description
1	TRINITY_DN8	0.06	−0.04	0.47		
2	comp38073_c	0.05	−0.02	0.56		
3	TRINITY_DN1	0.04	−0.02	0.82		
4	TRINITY_DN5	0.04	−0.07	0.74		
5	TRINITY_DN1	0.03	−0.05	0.86		
6	TRINITY_DN1	0.03	−0.04	0.89		
7	TRINITY_DN1	0.03	−0.02	0.58		
8	TRINITY_DN1	0.03	−0.02	0.87		
9	TRINITY_DN4	0.03	−0.03	0.7		
10	TRINITY_DN7	0.03	−0.04	0.76		
11	TRINITY_DN3	0.03	−0.03	0.61		
12	TRINITY_DN6	0.03	−0.03	0.9		
13	comp32732_c	0.03	−0.05	0.67		
14	TRINITY_DN9	0.03	−0.03	0.84		
15	TRINITY_DN1	0.03	−0.03	0.96		
16	TRINITY_DN3	0.03	−0.03	0.58		
17	comp9172_c0	0.03	−0.01	0.71		
18	TRINITY_DN3	0.03	−0.02	0.84		
19	TRINITY_DN6	0.03	−0.01	0.61		
20	TRINITY_DN1	0.03	−0.04	0.64		

Correlation Cluster

Spot Summary: E

metagenes = 131
genes = 1504

<r> metagenes = 0.94

beta: r2= 0.01 / log p= −0.22

samples with spot = 0 (0 %)

Overview Map

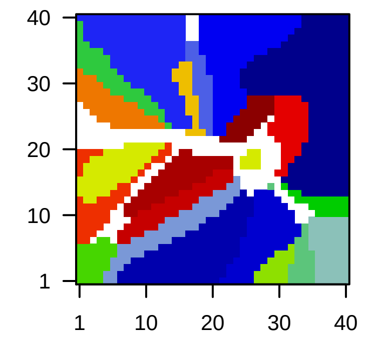

Spot

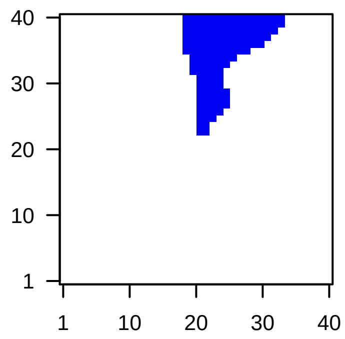

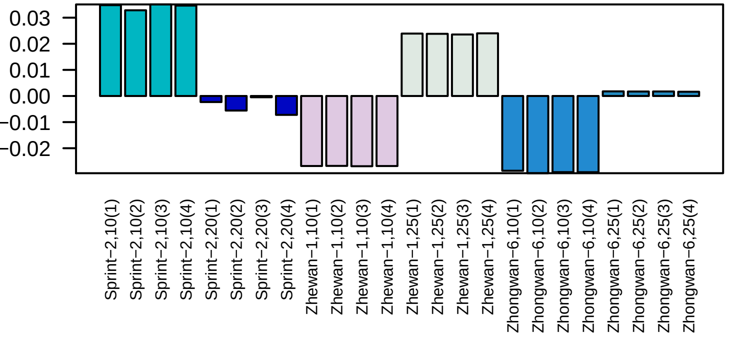

Spot Genelist

Rank	ID	max e	min e	r	Symbol	Description
1	TRINITY_DN1	0.53	−0.47	0.92		
2	TRINITY_DN8	0.48	−0.15	0.89		
3	TRINITY_DN1	0.46	−0.47	0.84		
4	TRINITY_DN2	0.45	−0.28	0.85		
5	TRINITY_DN4	0.44	−0.45	0.78		
6	TRINITY_DN4	0.42	−0.34	0.87		
7	TRINITY_DN5	0.41	−0.24	0.92		
8	TRINITY_DN3	0.41	−0.17	0.83		
9	TRINITY_DN1	0.41	−0.26	0.76		
10	TRINITY_DN6	0.38	−0.34	0.93		
11	TRINITY_DN4	0.38	−0.19	0.95		
12	TRINITY_DN9	0.38	−0.22	0.96		
13	TRINITY_DN2	0.38	−0.2	0.87		
14	TRINITY_DN3	0.37	−0.16	0.89		
15	TRINITY_DN5	0.37	−0.37	0.92		
16	TRINITY_DN6	0.36	−0.18	0.84		
17	comp35375_c	0.35	−0.13	0.9		
18	TRINITY_DN3	0.35	−0.31	0.93		
19	TRINITY_DN9	0.34	−0.41	0.92		
20	TRINITY_DN7	0.33	−0.26	0.88		

Correlation Cluster

Spot Summary: F

metagenes = 69
genes = 1299

<r> metagenes = 0.93

beta: r2= 0 / log p= −0.09

samples with spot = 0 (0 %)

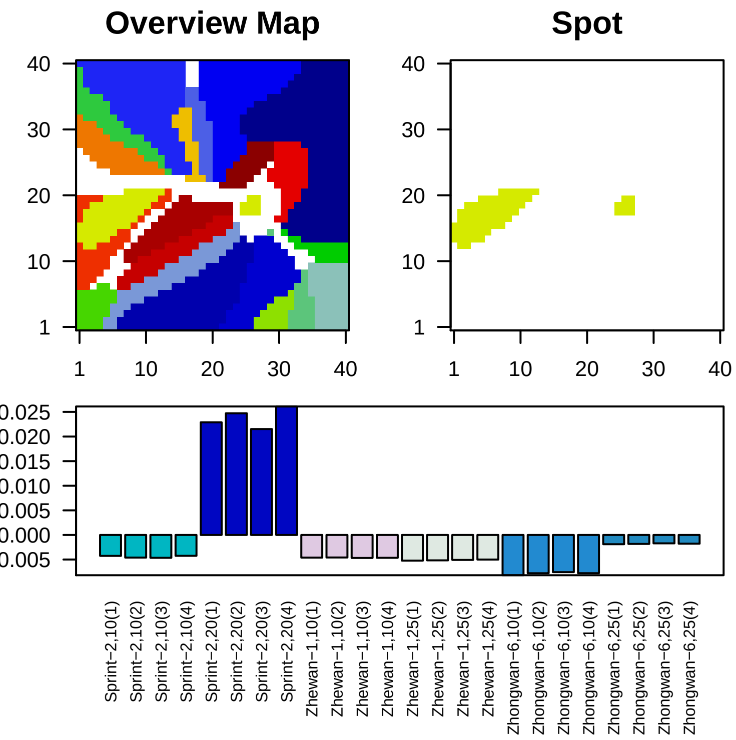

Spot Genelist

Rank	ID	max e	min e	r	Symbol	Description
1	TRINITY_DN3	0.35	−0.17	0.74		
2	comp12834_c	0.3	−0.09	0.9		
3	TRINITY_DN1	0.28	−0.1	0.84		
4	TRINITY_DN5	0.25	−0.08	0.73		
5	TRINITY_DN1	0.25	−0.03	0.81		
6	comp36918_c	0.25	−0.08	0.92		
7	TRINITY_DN4	0.24	−0.07	0.85		
8	TRINITY_DN1	0.23	−0.06	0.92		
9	TRINITY_DN2	0.22	−0.09	0.89		
10	TRINITY_DN1	0.22	−0.09	0.85		
11	TRINITY_DN4	0.21	−0.07	0.85		
12	TRINITY_DN8	0.21	−0.09	0.85		
13	comp41013_c	0.2	−0.09	0.78		
14	TRINITY_DN1	0.2	−0.04	0.95		
15	TRINITY_DN6	0.2	−0.09	0.83		
16	TRINITY_DN3	0.2	−0.06	0.95		
17	comp40907_c	0.19	−0.1	0.97		
18	TRINITY_DN5	0.19	−0.04	0.81		
19	TRINITY_DN1	0.19	−0.04	0.98		
20	TRINITY_DN2	0.19	−0.06	0.97		

Correlation Cluster

Spot Summary: G

metagenes = 51
genes = 565

<r> metagenes = 0.92
<r> genes = 0.63
beta: r2= 0.07 / log p= −0.64

samples with spot = 0 (0 %)

Overview Map

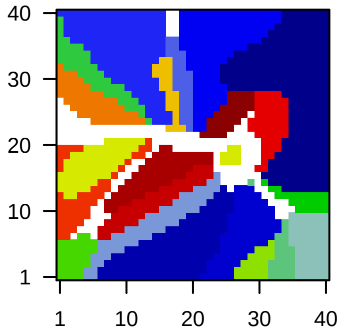

Spot

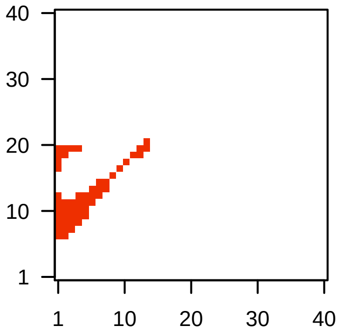

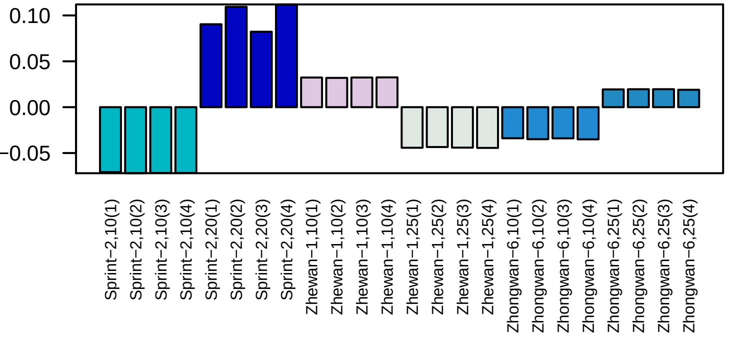

Spot Genelist

Rank	ID	max e	min e	r	Symbol	Description
1	TRINITY_DN1	1.36	−0.32	0.79		
2	TRINITY_DN1	1.24	−0.55	0.74		
3	TRINITY_DN1	1.1	−0.2	0.8		
4	TRINITY_DN2	1.01	−0.53	0.84		
5	TRINITY_DN4	0.93	−0.2	0.87		
6	TRINITY_DN9	0.79	−0.31	0.94		
7	TRINITY_DN5	0.78	−0.52	0.95		
8	TRINITY_DN1	0.71	−0.32	0.91		
9	TRINITY_DN1	0.69	−0.27	0.97		
10	TRINITY_DN1	0.68	−0.33	0.78		
11	TRINITY_DN1	0.67	−0.39	0.97		
12	TRINITY_DN1	0.65	−0.12	0.82		
13	TRINITY_DN4	0.62	−0.68	0.76		
14	TRINITY_DN1	0.62	−0.53	0.94		
15	TRINITY_DN3	0.62	−0.68	0.77		
16	TRINITY_DN1	0.61	−0.29	0.82		
17	TRINITY_DN3	0.6	−0.22	0.72		
18	TRINITY_DN8	0.59	−0.4	0.88		
19	TRINITY_DN1	0.57	−0.19	0.89		
20	TRINITY_DN5	0.56	−0.14	0.55		

Correlation Cluster

Spot Summary: H

metagenes = 50
genes = 921

<r> metagenes = 0.97
<r> genes = 0.68
beta: r2= 0 / log p= −0.1

samples with spot = 0 (0 %)

Spot Genelist

Rank	ID	max e	min e	r	Symbol	Description
1	TRINITY_DN4	0.38	−0.13	0.93		
2	TRINITY_DN1	0.32	−0.1	0.96		
3	TRINITY_DN3	0.28	−0.15	0.8		
4	comp26858_c	0.27	−0.05	0.98		
5	comp41036_c	0.24	−0.04	0.99		
6	TRINITY_DN2	0.24	−0.06	0.98		
7	TRINITY_DN8	0.24	−0.07	0.99		
8	TRINITY_DN2	0.24	−0.06	0.98		
9	TRINITY_DN3	0.22	−0.06	0.99		
10	TRINITY_DN2	0.22	−0.1	0.77		
11	TRINITY_DN7	0.22	−0.11	0.79		
12	TRINITY_DN1	0.2	−0.07	0.96		
13	TRINITY_DN4	0.2	−0.08	0.96		
14	TRINITY_DN5	0.19	−0.05	0.99		
15	comp39466_c	0.19	−0.04	0.98		
16	TRINITY_DN4	0.19	−0.05	0.97		
17	TRINITY_DN4	0.19	−0.09	0.87		
18	TRINITY_DN2	0.18	−0.07	0.98		
19	TRINITY_DN1	0.17	−0.07	0.91		
20	TRINITY_DN8	0.17	−0.04	0.9		

Overview Map

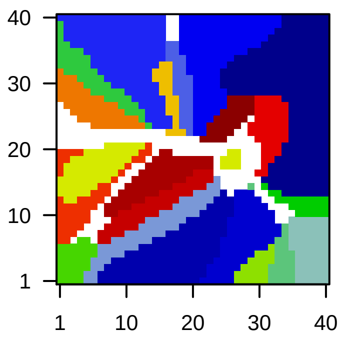

Spot

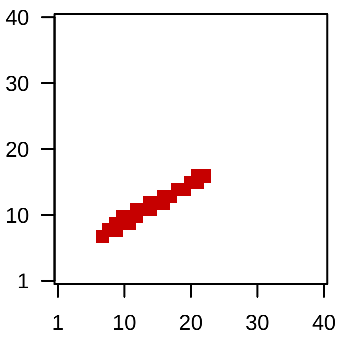

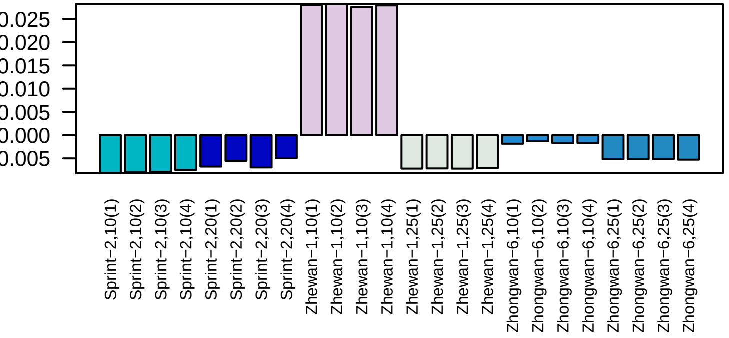

Correlation Cluster

Spot Summary: I

metagenes = 61
genes = 1302

<r> metagenes = 0.92

beta: r2= 0 / log p= −0.04

samples with spot = 0 (0 %)

Overview Map

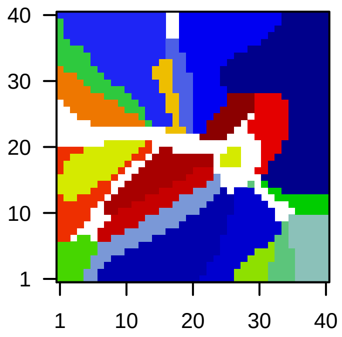

Spot

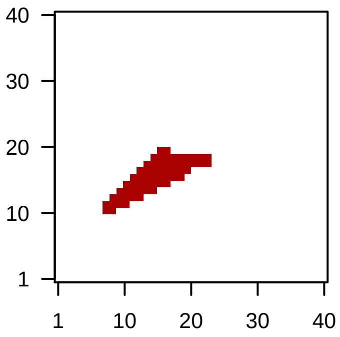

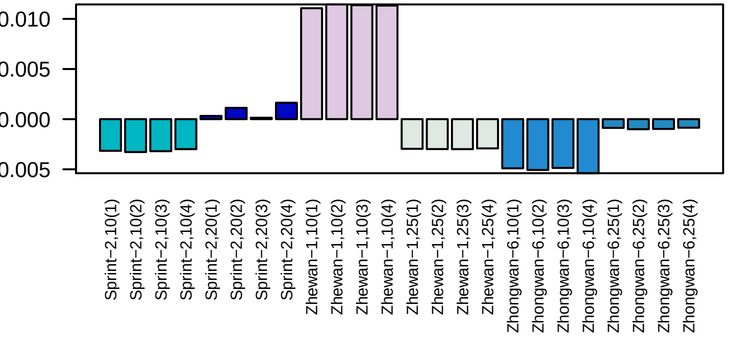

Spot Genelist

Rank	ID	max e	min e	r	Symbol	Description
1	TRINITY_DN1	0.12	−0.13	0.76		
2	TRINITY_DN4	0.12	−0.03	0.7		
3	TRINITY_DN4	0.09	−0.07	0.9		
4	TRINITY_DN7	0.08	−0.06	0.83		
5	TRINITY_DN1	0.07	−0.02	0.95		
6	TRINITY_DN1	0.07	−0.04	0.9		
7	TRINITY_DN5	0.07	−0.06	0.93		
8	TRINITY_DN8	0.07	−0.04	0.86		
9	TRINITY_DN1	0.06	−0.04	0.98		
10	TRINITY_DN7	0.06	−0.04	0.86		
11	TRINITY_DN2	0.06	−0.04	0.89		
12	TRINITY_DN3	0.06	−0.04	0.94		
13	TRINITY_DN5	0.06	−0.03	0.8		
14	TRINITY_DN2	0.06	−0.03	0.95		
15	TRINITY_DN1	0.06	−0.05	0.85		
16	TRINITY_DN7	0.06	−0.03	0.84		
17	TRINITY_DN4	0.06	−0.03	0.97		
18	TRINITY_DN1	0.06	−0.02	0.97		
19	TRINITY_DN1	0.06	−0.03	0.78		
20	TRINITY_DN1	0.06	−0.03	0.92		

Correlation Cluster

Spot Summary: J

metagenes = 32
genes = 173

<r> metagenes = 0.96
<r> genes = 0.65
beta: r2= 5.86 / log p= −Inf

samples with spot = 16 (66.7 %)
Sprint−2(20) : 4 (100 %)
Zhewan−1(10) : 4 (100 %)
Zhongwan−6(10) : 4 (100 %)
Zhongwan−6(25) : 4 (100 %)

Overview Map

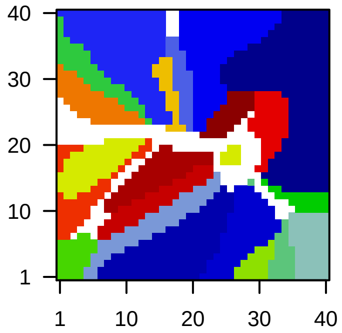

Spot

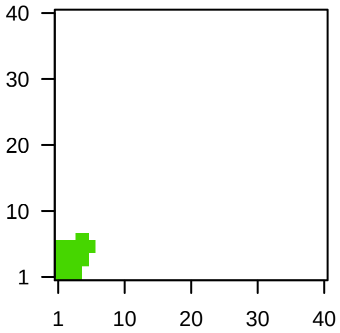

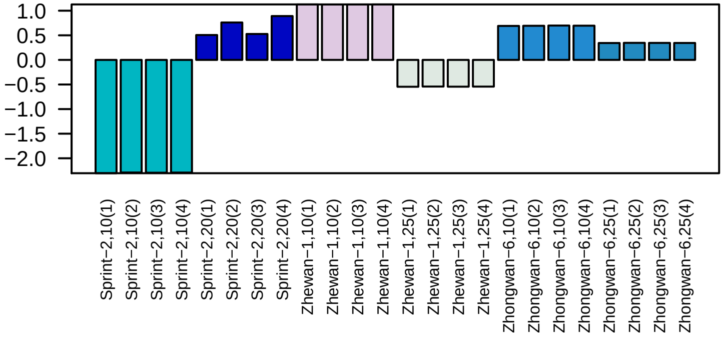

Spot Genelist

Rank	ID	max e	min e	r	Symbol	Description
1	TRINITY_DN2	23.2	−19.57	0.81		
2	TRINITY_DN1	21.33	−106.92	0.89		
3	TRINITY_DN7	20.26	−13.18	0.81		
4	TRINITY_DN4	19.94	−13.52	0.6		
5	TRINITY_DN3	16.43	−9.68	0.63		
6	TRINITY_DN1	13.69	−6.37	0.71		
7	TRINITY_DN7	13.41	−12.69	0.91		
8	TRINITY_DN3	13.38	−29.37	0.87		
9	TRINITY_DN2	11.58	−8.49	0.78		
10	TRINITY_DN3	10.97	−5.32	0.57		
11	TRINITY_DN3	9.96	−9.05	0.54		
12	TRINITY_DN7	7.7	−5.96	0.76		
13	TRINITY_DN3	5.8	−7.86	0.73		
14	TRINITY_DN3	5.67	−6.93	0.76		
15	TRINITY_DN3	5.25	−4.21	0.64		
16	TRINITY_DN3	4.83	−3.23	0.71		
17	TRINITY_DN3	4.13	−3.93	0.78		
18	TRINITY_DN1	4.04	−4.03	0.77		
19	TRINITY_DN6	3.63	−3.54	0.91		
20	TRINITY_DN1	3.44	−2.28	0.81		

Correlation Cluster

Spot Summary: K

metagenes = 154
genes = 2097

<r> metagenes = 0.91

beta: r2= 0.03 / log p= −0.34

samples with spot = 0 (0 %)

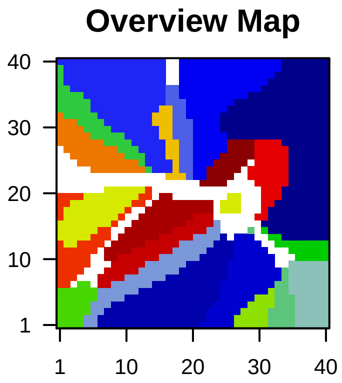

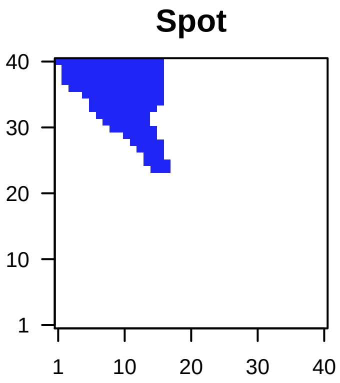

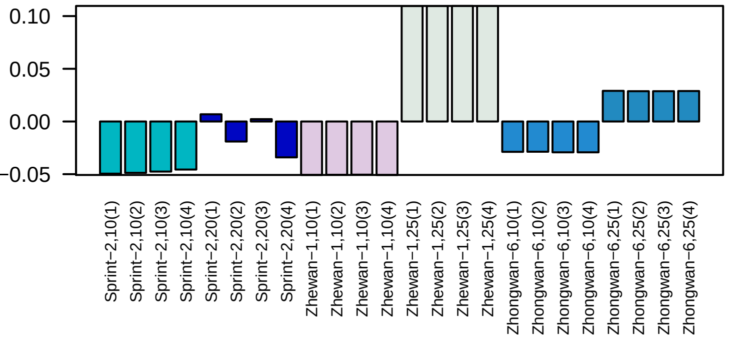

Spot Genelist

Rank	ID	max e	min e	r	Symbol	Description
1	TRINITY_DN2	19.02	−23.73	0.84		
2	TRINITY_DN6	13.45	−12.66	0.9		
3	TRINITY_DN2	10.34	−9.73	0.85		
4	TRINITY_DN4	10.09	−9.97	0.86		
5	TRINITY_DN4	9.16	−4.51	0.92		
6	TRINITY_DN2	8.02	−4.57	0.99		
7	TRINITY_DN2	7.51	−8.63	0.84		
8	TRINITY_DN4	6.31	−3.15	0.96		
9	comp39373_c	6.03	−3.43	0.99		
10	TRINITY_DN2	3.51	−1.01	0.96		
11	TRINITY_DN3	2.68	−3.01	0.59		
12	TRINITY_DN1	2.46	−0.77	0.91		
13	TRINITY_DN3	2.45	−1.45	0.99		
14	TRINITY_DN1	2.42	−1.79	0.82		
15	TRINITY_DN1	2.2	−0.88	0.87		
16	TRINITY_DN1	1.98	−1.06	0.7		
17	TRINITY_DN3	1.73	−0.78	0.98		
18	TRINITY_DN2	1.69	−1.29	0.88		
19	TRINITY_DN7	1.61	−1.45	0.8		
20	TRINITY_DN5	1.61	−0.68	0.87		

Correlation Cluster

Spot Summary: L

metagenes = 49
genes = 673

<r> metagenes = 0.96
<r> genes = 0.64
beta: r2= 0.02 / log p= −0.31

samples with spot = 0 (0 %)

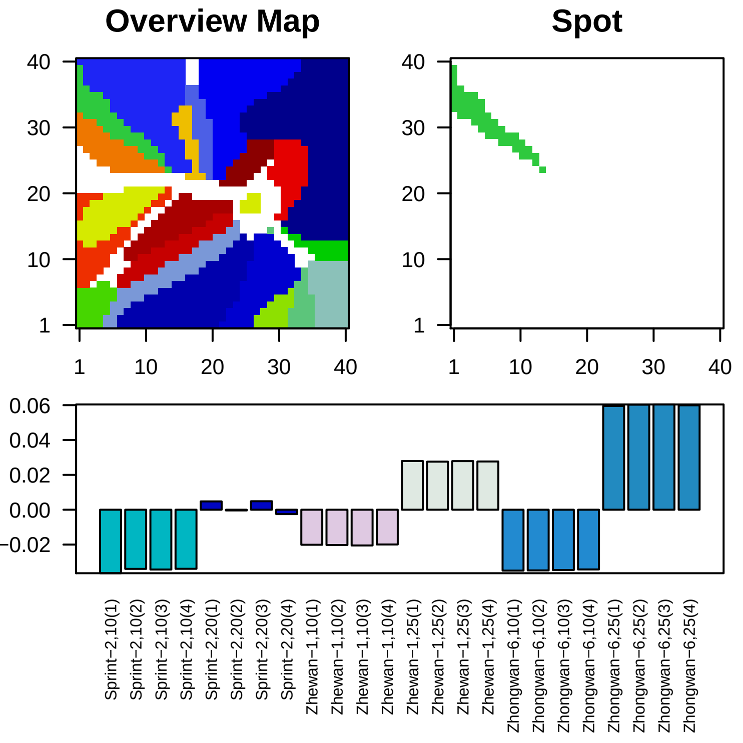

Spot Genelist

Rank	ID	max e	min e	r	Symbol	Description
1	TRINITY_DN1	1.4	−0.89	0.92		
2	TRINITY_DN2	1.37	−0.4	0.9		
3	TRINITY_DN4	1.08	−0.63	0.98		
4	TRINITY_DN1	0.97	−0.43	0.95		
5	TRINITY_DN3	0.92	−1.14	0.86		
6	TRINITY_DN3	0.86	−0.5	0.88		
7	TRINITY_DN2	0.74	−0.86	0.66		
8	TRINITY_DN7	0.65	−0.54	0.87		
9	TRINITY_DN1	0.63	−0.43	0.88		
10	TRINITY_DN6	0.61	−0.33	0.91		
11	TRINITY_DN8	0.6	−0.35	0.74		
12	TRINITY_DN3	0.57	−0.37	0.94		
13	TRINITY_DN4	0.57	−0.22	0.91		
14	comp39331_c	0.55	−0.52	0.69		
15	TRINITY_DN4	0.55	−0.42	0.88		
16	TRINITY_DN3	0.53	−0.45	0.95		
17	TRINITY_DN1	0.51	−0.45	0.85		
18	TRINITY_DN4	0.51	−0.66	0.71		
19	TRINITY_DN7	0.5	−0.15	0.76		
20	TRINITY_DN5	0.49	−0.23	0.92		

Correlation Cluster

Spot Summary: M

metagenes = 23
genes = 604

<r> metagenes = 0.98
<r> genes = 0.65
beta: r2= 0 / log p= −0.03

samples with spot = 0 (0 %)

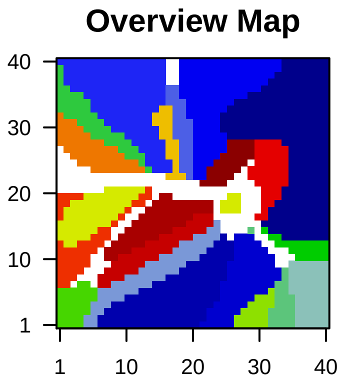

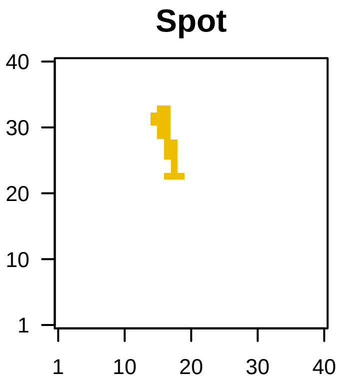

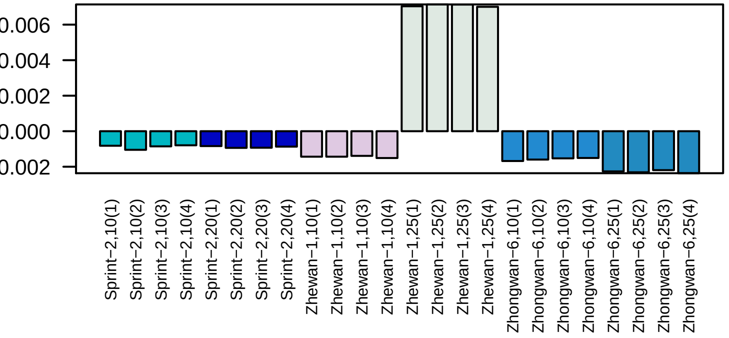

Spot Genelist

Rank	ID	max e	min e	r	Symbol	Description
1	TRINITY_DN6	0.03	0	0.57		
2	comp28566_c	0.02	0	0.76		
3	TRINITY_DN1	0.02	0	0.93		
4	TRINITY_DN5	0.02	−0.01	0.84		
5	TRINITY_DN8	0.02	−0.02	0.55		
6	TRINITY_DN5	0.02	−0.01	0.91		
7	TRINITY_DN4	0.02	0	0.77		
8	TRINITY_DN2	0.02	0	0.91		
9	TRINITY_DN8	0.02	−0.02	0.53		
10	comp41609_c	0.02	−0.01	0.68		
11	comp21494_c	0.02	0	0.93		
12	comp31877_c	0.02	−0.01	0.73		
13	comp61810_c	0.02	0	0.93		
14	TRINITY_DN1	0.02	−0.01	0.84		
15	TRINITY_DN1	0.02	−0.01	0.89		
16	TRINITY_DN1	0.02	−0.01	0.52		
17	comp47480_c	0.02	0	0.93		
18	TRINITY_DN4	0.02	0	0.94		
19	TRINITY_DN5	0.02	−0.02	0.85		
20	TRINITY_DN4	0.02	−0.01	0.87		

Correlation Cluster

Spot Summary: N

metagenes = 31
genes = 495

<r> metagenes = 0.96
<r> genes = 0.61
beta: r2= 0 / log p= −0.04

samples with spot = 0 (0 %)

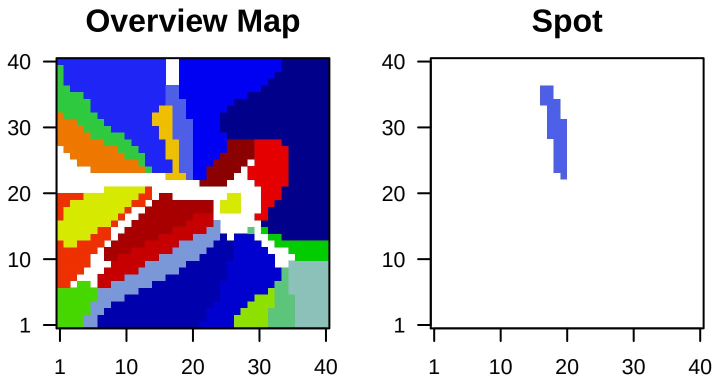

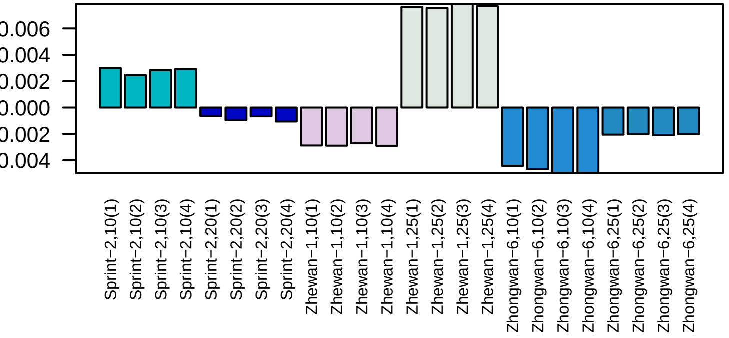

Spot Genelist

Rank	ID	max e	min e	r	Symbol	Description
1	TRINITY_DN1	0.04	−0.02	0.47		
2	TRINITY_DN4	0.03	−0.03	0.84		
3	TRINITY_DN1	0.03	−0.04	0.81		
4	TRINITY_DN1	0.03	−0.01	0.49		
5	TRINITY_DN8	0.03	−0.03	0.53		
6	TRINITY_DN1	0.03	−0.03	0.91		
7	TRINITY_DN3	0.03	−0.02	0.8		
8	TRINITY_DN1	0.03	−0.01	0.56		
9	TRINITY_DN5	0.03	−0.01	0.9		
10	TRINITY_DN1	0.02	−0.04	0.55		
11	TRINITY_DN4	0.02	−0.02	0.81		
12	TRINITY_DN3	0.02	−0.03	0.65		
13	TRINITY_DN6	0.02	−0.02	0.75		
14	TRINITY_DN8	0.02	−0.01	0.88		
15	comp35196_c	0.02	−0.01	0.62		
16	TRINITY_DN1	0.02	−0.02	0.82		
17	TRINITY_DN4	0.02	−0.01	0.87		
18	TRINITY_DN9	0.02	−0.02	0.71		
19	TRINITY_DN3	0.02	−0.02	0.52		
20	TRINITY_DN7	0.02	−0.01	0.86		

Correlation Cluster

Spot Summary: O

metagenes = 80
genes = 2804

<r> metagenes = 0.95

beta: r2= 0 / log p= −0.02

samples with spot = 0 (0 %)

Spot Genelist

Rank	ID	max e	min e	r	Symbol	Description
1	TRINITY_DN1	0.09	−0.02	0.68		
2	TRINITY_DN7	0.07	−0.05	0.88		
3	TRINITY_DN6	0.07	−0.06	0.65		
4	TRINITY_DN3	0.07	−0.01	0.42		
5	TRINITY_DN7	0.07	−0.02	0.86		
6	comp18398_c	0.07	−0.03	0.82		
7	TRINITY_DN3	0.07	−0.03	0.87		
8	comp40778_c	0.06	−0.01	0.64		
9	TRINITY_DN5	0.06	−0.02	0.75		
10	TRINITY_DN2	0.06	−0.04	0.82		
11	TRINITY_DN1	0.06	−0.02	0.79		
12	TRINITY_DN4	0.06	−0.01	0.63		
13	TRINITY_DN6	0.06	−0.04	0.81		
14	TRINITY_DN3	0.06	−0.02	0.59		
15	TRINITY_DN3	0.06	−0.05	0.8		
16	TRINITY_DN8	0.06	−0.05	0.57		
17	TRINITY_DN4	0.06	−0.05	0.86		
18	TRINITY_DN6	0.05	−0.03	0.9		
19	TRINITY_DN1	0.05	−0.05	0.87		
20	TRINITY_DN3	0.05	−0.02	0.95		

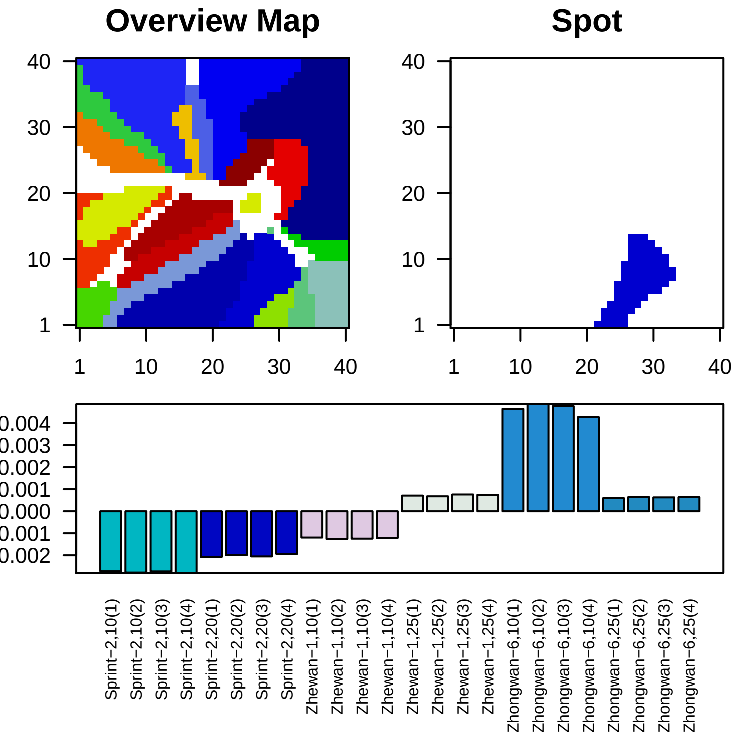

Correlation Cluster

Spot Summary: P

metagenes = 132
genes = 2763

<r> metagenes = 0.93

beta: r2= 0.01 / log p= −0.22

samples with spot = 0 (0 %)

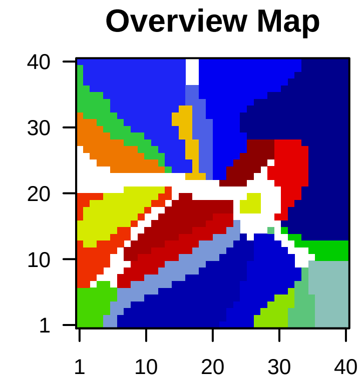

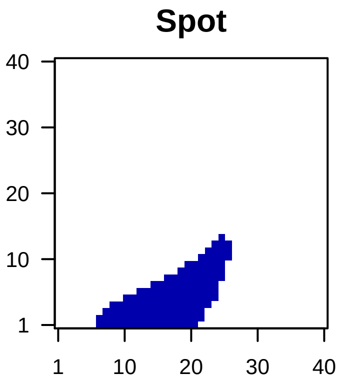

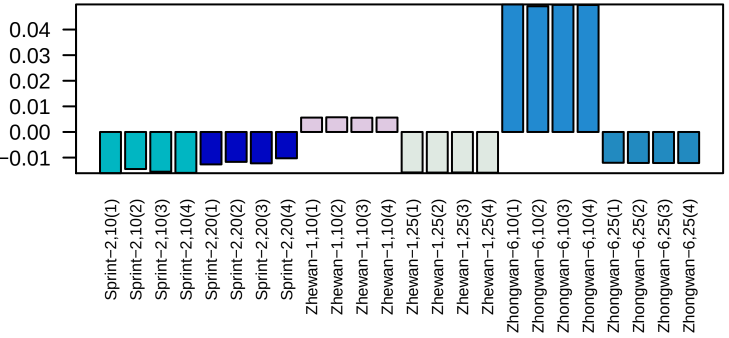

Spot Genelist

Rank	ID	max e	min e	r	Symbol	Description
1	TRINITY_DN4	0.88	−0.2	0.95		
2	TRINITY_DN5	0.82	−0.19	0.98		
3	TRINITY_DN4	0.81	−0.2	0.99		
4	TRINITY_DN3	0.78	−0.16	0.95		
5	TRINITY_DN6	0.78	−0.3	0.84		
6	TRINITY_DN7	0.74	−0.16	0.97		
7	TRINITY_DN6	0.61	−0.3	0.85		
8	TRINITY_DN2	0.6	−0.15	0.96		
9	TRINITY_DN4	0.57	−0.1	0.95		
10	TRINITY_DN2	0.53	−0.27	0.55		
11	TRINITY_DN5	0.53	−0.12	0.95		
12	TRINITY_DN5	0.52	−0.24	0.81		
13	TRINITY_DN4	0.52	−0.19	0.94		
14	TRINITY_DN6	0.5	−0.1	0.83		
15	TRINITY_DN2	0.5	−0.33	0.69		
16	TRINITY_DN3	0.49	−0.12	0.99		
17	TRINITY_DN2	0.48	−0.22	0.94		
18	TRINITY_DN6	0.47	−0.27	0.85		
19	TRINITY_DN6	0.47	−0.29	0.9		
20	TRINITY_DN1	0.47	−0.13	0.99		

Correlation Cluster

Spot Summary: Q

metagenes = 66
genes = 1123

<r> metagenes = 0.96

beta: r2= 0.02 / log p= −0.33

samples with spot = 0 (0 %)

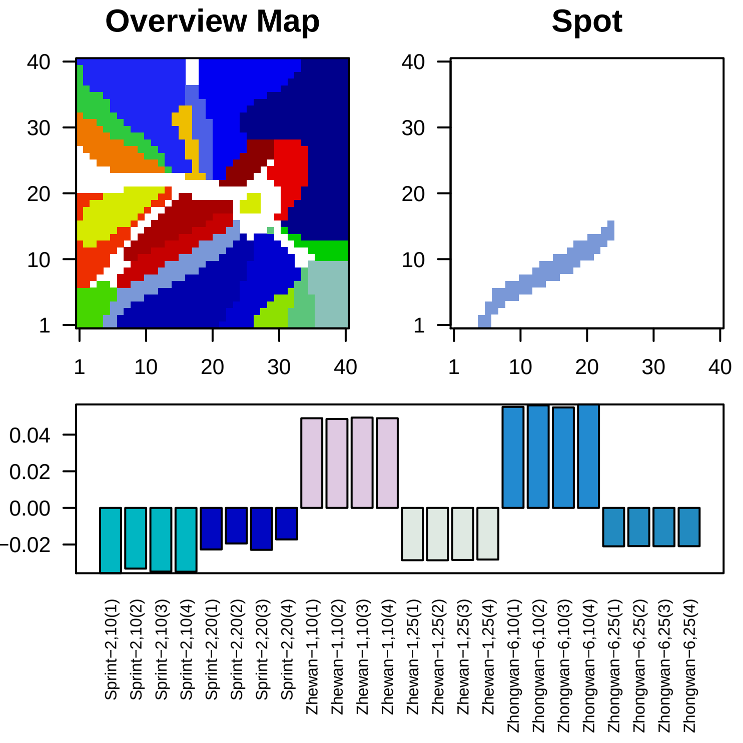

Spot Genelist

Rank	ID	max e	min e	r	Symbol	Description
1	TRINITY_DN4	1.56	−0.78	0.72		
2	comp18180_c	1.54	−0.53	0.92		
3	TRINITY_DN7	1.25	−0.29	0.71		
4	TRINITY_DN8	1.23	−0.34	0.74		
5	TRINITY_DN5	1.22	−0.45	0.96		
6	comp14844_c	1.17	−0.6	0.95		
7	TRINITY_DN1	1.08	−0.66	0.88		
8	TRINITY_DN2	1.07	−0.26	0.75		
9	TRINITY_DN1	1.04	−0.41	0.98		
10	TRINITY_DN1	0.95	−0.38	0.81		
11	TRINITY_DN1	0.94	−0.2	0.76		
12	TRINITY_DN1	0.9	−0.42	0.98		
13	TRINITY_DN4	0.89	−0.45	0.93		
14	TRINITY_DN2	0.88	−0.32	0.91		
15	TRINITY_DN3	0.87	−0.42	0.99		
16	TRINITY_DN6	0.86	−0.65	0.96		
17	comp36789_c	0.86	−0.48	0.86		
18	TRINITY_DN9	0.85	−0.36	0.99		
19	TRINITY_DN4	0.83	−0.21	0.82		
20	TRINITY_DN3	0.83	−0.45	0.57		

Correlation Cluster

Spot Summary: R

metagenes = 55
genes = 739

<r> metagenes = 0.95
<r> genes = 0.57
beta: r2= 0 / log p= −0.04

samples with spot = 0 (0 %)

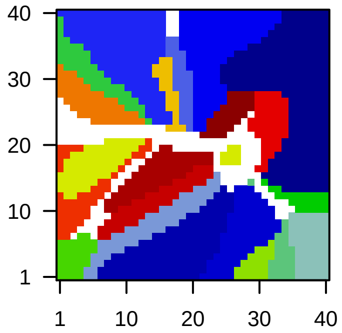

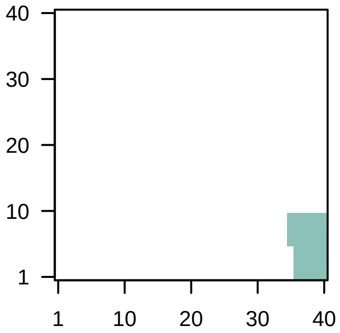

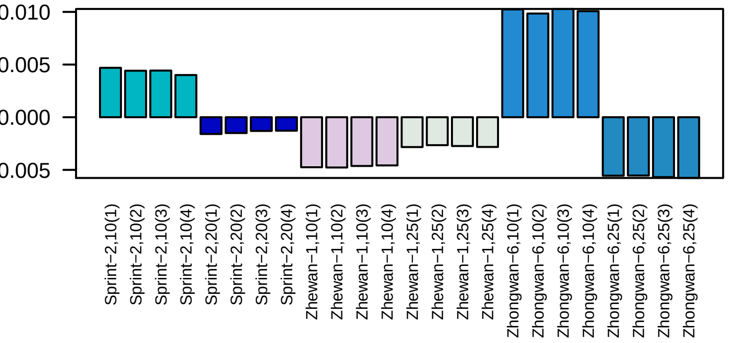

Spot Genelist

Rank	ID	max e	min e	r	Symbol	Description
1	TRINITY_DN1	0.07	−0.06	0.8		
2	TRINITY_DN6	0.07	−0.04	0.89		
3	TRINITY_DN3	0.07	−0.05	0.69		
4	TRINITY_DN4	0.06	−0.03	0.77		
5	TRINITY_DN2	0.06	−0.06	0.46		
6	TRINITY_DN9	0.06	−0.04	0.86		
7	TRINITY_DN4	0.06	−0.01	0.55		
8	TRINITY_DN3	0.06	−0.05	0.81		
9	TRINITY_DN3	0.05	−0.03	0.94		
10	TRINITY_DN5	0.05	−0.03	0.73		
11	TRINITY_DN2	0.05	−0.02	0.79		
12	TRINITY_DN6	0.05	−0.03	0.92		
13	TRINITY_DN7	0.05	−0.03	0.73		
14	TRINITY_DN1	0.05	−0.03	0.87		
15	TRINITY_DN4	0.05	−0.02	0.72		
16	TRINITY_DN9	0.04	−0.03	0.9		
17	TRINITY_DN1	0.04	−0.04	0.67		
18	TRINITY_DN1	0.04	−0.03	0.52		
19	TRINITY_DN1	0.04	−0.04	0.8		
20	TRINITY_DN6	0.04	−0.02	0.95		

Correlation Cluster

Spot Summary: S

metagenes = 25
genes = 709

<r> metagenes = 0.96
<r> genes = NA
beta: r2= 0 / log p= −0.02

samples with spot = 0 (0 %)

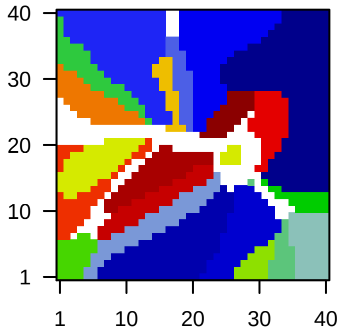

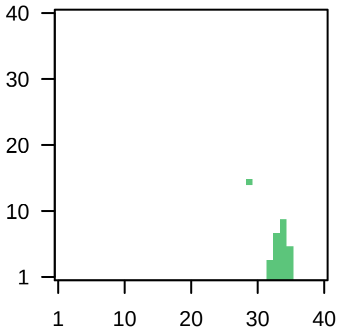

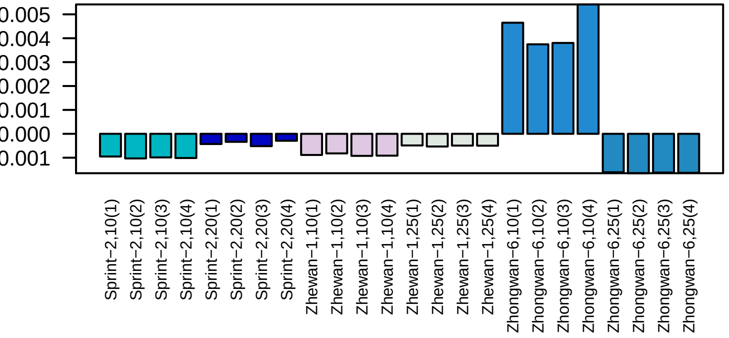

Spot Genelist

Rank	ID	max e	min e	r	Symbol	Description
1	TRINITY_DN‹	0.05	0	0.51		
2	TRINITY_DN‹	0.05	0	0.56		
3	TRINITY_DN‹	0.04	0	0.48		
4	TRINITY_DN‹	0.04	0	0.6		
5	comp49596_c	0.04	0	0.55		
6	comp56826_c	0.03	0	0.75		
7	comp44207_c	0.03	0	0.56		
8	TRINITY_DN‹	0.03	0	0.62		
9	TRINITY_DN‹	0.03	0	0.81		
10	TRINITY_DN‹	0.03	0	0.28		
11	TRINITY_DN‹	0.03	−0.01	0.87		
12	TRINITY_DN‹	0.03	0	0.81		
13	TRINITY_DN‹	0.03	0	0.6		
14	TRINITY_DN‹	0.03	0	0.72		
15	TRINITY_DN‹	0.02	−0.02	0.66		
16	TRINITY_DN‹	0.02	0	0.67		
17	TRINITY_DN‹	0.02	−0.02	0.53		
18	TRINITY_DN‹	0.02	−0.01	0.38		
19	comp48313_c	0.02	0	0.62		
20	TRINITY_DN‹	0.02	0	0.96		

Correlation Cluster

Spot Summary: T

metagenes = 22
genes = 365

<r> metagenes = 0.98
<r> genes = 0.59
beta: r2= 0 / log p= −0.04

samples with spot = 0 (0 %)

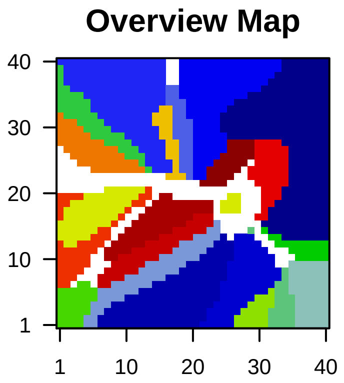

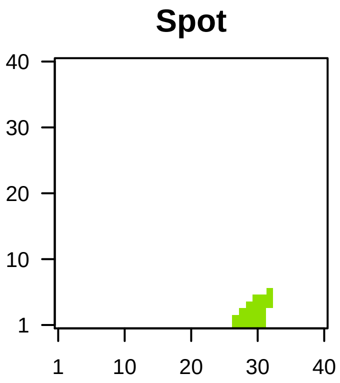

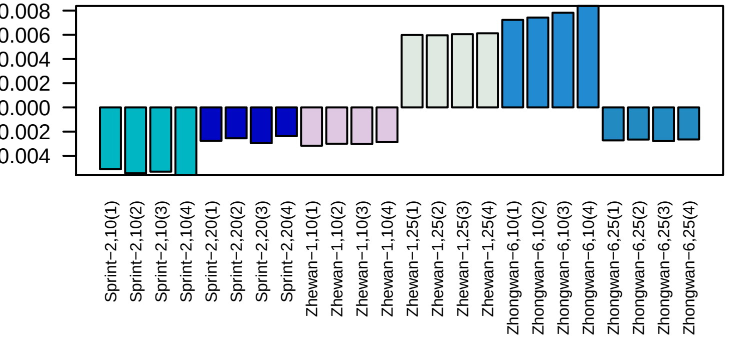

Spot Genelist

Rank	ID	max e	min e	r	Symbol	Description
1	comp49276_c	0.04	0	0.48		
2	TRINITY_DN6	0.04	−0.01	0.65		
3	TRINITY_DN1	0.04	−0.01	0.55		
4	TRINITY_DN8	0.04	−0.01	0.67		
5	TRINITY_DN1	0.04	0	0.43		
6	TRINITY_DN1	0.04	−0.02	0.79		
7	TRINITY_DN1	0.03	−0.03	0.69		
8	comp40310_c	0.03	−0.01	0.5		
9	TRINITY_DN5	0.03	−0.02	0.72		
10	TRINITY_DN1	0.03	−0.02	0.61		
11	TRINITY_DN3	0.03	−0.02	0.83		
12	TRINITY_DN3	0.03	−0.01	0.85		
13	TRINITY_DN3	0.03	−0.02	0.77		
14	TRINITY_DN1	0.03	−0.01	0.59		
15	TRINITY_DN2	0.03	0	0.65		
16	TRINITY_DN1	0.03	−0.02	0.81		
17	TRINITY_DN7	0.03	−0.02	0.79		
18	TRINITY_DN1	0.03	−0.01	0.79		
19	TRINITY_DN1	0.03	−0.02	0.63		
20	TRINITY_DN1	0.03	−0.01	0.62		

Correlation Cluster

Spot Summary: U

metagenes = 53
genes = 1078

<r> metagenes = 0.96

beta: r2= 0 / log p= −0.08

samples with spot = 0 (0 %)

Overview Map

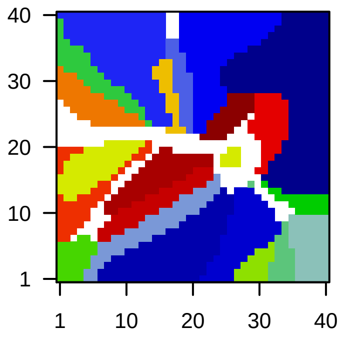

Spot

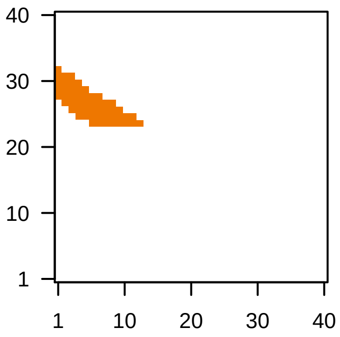

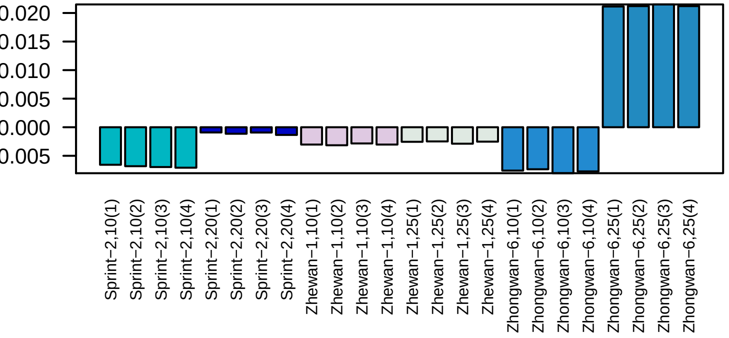

Spot Genelist

Rank	ID	max e	min e	r	Symbol	Description
1	TRINITY_DN9	0.19	−0.1	0.92		
2	TRINITY_DN1	0.18	−0.09	0.94		
3	TRINITY_DN2	0.18	−0.04	0.97		
4	TRINITY_DN8	0.17	−0.11	0.8		
5	TRINITY_DN3	0.17	−0.06	0.95		
6	TRINITY_DN7	0.16	−0.12	0.71		
7	TRINITY_DN4	0.16	−0.03	0.97		
8	TRINITY_DN5	0.15	−0.05	0.96		
9	TRINITY_DN3	0.15	−0.15	0.39		
10	TRINITY_DN8	0.15	−0.07	0.93		
11	TRINITY_DN6	0.15	−0.07	0.96		
12	TRINITY_DN2	0.14	−0.08	0.74		
13	comp40946_c	0.14	−0.03	0.95		
14	TRINITY_DN2	0.13	−0.07	0.63		
15	TRINITY_DN3	0.13	−0.13	0.59		
16	TRINITY_DN8	0.13	−0.08	0.68		
17	TRINITY_DN6	0.13	−0.1	0.6		
18	TRINITY_DN1	0.13	−0.15	0.8		
19	TRINITY_DN6	0.13	−0.06	0.94		
20	TRINITY_DN1	0.12	−0.04	0.97		
